# GreenFruitDetector: Lightweight green fruit detector in orchard environment

**DOI:** 10.1371/journal.pone.0312164

**Published:** 2024-11-14

**Authors:** Jing Wang, Yu Shang, Xiuling Zheng, Peng Zhou, Shiyue Li, Huan Wang

**Affiliations:** 1 School of Computer Science and Engineering, North China Institute of Aerospace Engineering, Langfang, China; 2 School of Information Science and Engineering, Xinjiang College of Science & Technology, Korla, China; Bahria University - Lahore Campus, PAKISTAN

## Abstract

Detecting green fruits presents significant challenges due to their close resemblance in color to the leaves in an orchard environment. We designed GreenFruitDetector, a lightweight model based on an improved YOLO v8 architecture, specifically for green fruit detection. In the Backbone network, we replace ordinary convolution with Deformable Convolution to enhance the extraction of geometric features. Additionally, we designed MCAG-DC (Multi-path Coordinate Attention Guided Deformer Convolution) to replace the convolution in C2f, enhancing the Backbone’s feature extraction capability when encountering occlusion problems. For the Neck part of the algorithm, we designed a Fusion-neck structure that integrates spatial detail information from feature maps at different scales, thereby enhancing the network’s ability to extract multi-scale information. Additionally, we devised a new detection head that incorporates multi-scale information, significantly improving the detection of small and distant objects. Finally, we applied channel pruning techniques to reduce the model size, parameter count, and FLOPs to 50%, 55%, and 44% of the original, respectively. We trained and evaluated the improved model on three green fruit datasets. The accuracy of the improved model reached 94.5%, 84.4%, and 85.9% on the Korla Pear, Guava, and Green Apple datasets, respectively, representing improvements of 1.17%, 1.1%, and 1.77% over the baseline model. The mAP@0.5 increased by 0.72%, 6.5%, and 0.9%, respectively, and the recall rate increased by 1.97%, 1.1%, and 0.49%, respectively.

## 1. Introduction

With recent advancements in artificial intelligence, particularly in vision-based intelligent systems, fruit-picking robots have gained enhanced capabilities for accurate fruit classification and detection. However, some types of fruits, like Fragrant pear, green apple, green mango, and guavas, pose challenges for vision-based picking robots because their colors closely resemble those of the leaves. This similarity poses difficulties in accurate classification and detection, leading to misclassification and missed detections, especially in orchard environments where factors like occlusion from other fruits or leaves, and distant small object further complicate detection tasks [1].

Designing vision-based picking robots that emulate human visual perception and judgment is a goal that researchers continuously strive to achieve. As deep learning technology continues to advance, deep learning-based object detection methods are also evolving rapidly, becoming a new research hotspot [2–7]. Current methods for automated fruit detection and identification are broadly categorized into two types: region proposal-based two-stage detection [8–10] and regression-based one-stage detection [11–16]. To address the specific challenge of distinguishing green fruits, researchers have introduced attention mechanisms [17–19], Multiscale Context Aggregation (MSCA) modules [20], and Focal Bottleneck Transformer modules [21] to enhance feature extraction capabilities. These methods all utilize convolutional layers for feature extraction. However, convolutional neural networks (CNNs) inherently struggle to model geometric transformations due to the fixed geometric structures in their building modules. Particularly, when fruits closely resemble leaves and are difficult to distinguish by color, it becomes necessary to extract the geometric structures of fruits and leaves. This enhanced geometric information can improve semantic understanding and recognition accuracy. DC (Deformer Convolution) [22] and DSC (Dynamic Snake Convolution) [23], which dynamically learn offsets to enhance CNNs’ transformation modeling capability, can improve the model’s ability to extract geometric features, and can also effectively enhance the detection accuracy of green fruits. However, there is still limited research in this area.

In intelligent harvesting, detection models must balance accuracy with the constraints of storage space and computational power on terminal devices. Designing lightweight models is also essential work. Consequently, the development of lightweight models becomes imperative. The lightweight solutions for intelligent harvesting typically include two categories: structural design and model compression. Structural design typically involves the incorporation of lightweight architectures, such as MobileNet, ShuffleNet, and GhostNet, into the model [24, 25]. Another common approach to model compression involves pruning techniques, specifically channel pruning [26, 27], to trim down the model.

In this study, we introduce the GreenFruitDetector, a lightweight model for detecting green fruits to address the challenge of distinguishing between fruits and leaves due to their similar colors in orchard environments. Our model is an improvement upon YOLOv8. To enhance the extraction of geometric features, especially when occluded, we propose Multi-path Coordinate Attention guided Deformer Convolution (MCAG-DC), which integrates spatial information and global context to guide the offsets of DC and integrates it into the Backbone of YOLO. Moreover, to address the challenge of detecting fruits at various scales in orchards, we propose Fusion-neck, fusing multi-scale feature in Neck, along with the Multiscale Small Object Detector (MSOD) to improve model accuracy. Additionally, for model lightweighting, we employ channel pruning, significantly reducing model size and accelerating inference time. We tested our model on three fruits with color similarity between leaves and fruits: pears, green apples, and guavas. Compared to several SOTA lightweight object detection models, our model excels in efficiency and real-time performance, as well as metrics such as Precision, Recall, and mAP@0.5. The main contributions of our research are outlined below:

1. In the Backbone, we utilized deformer convolution to replace the standard convolution, enhancing the model’s ability to extract geometric structures of fruits and leaves. Additionally, we designed a novel MCAG-DC, which calculates Multi-path Coordinate Attention to guide the deformer’s offsets. This approach enhances the Backbone’s feature extraction capability, particularly when encountering occlusion problems.
2. We proposed a Fusion-neck architecture, which introduces a Triple Feature Encoder (TFE) module and a Scale Sequence Feature Fusion Module (SSFF). These modules integrate spatial detail information from feature maps at three different scales, thereby enhancing the network’s ability to extract multi-scale information. Furthermore, a new detection head was designed to incorporate multi-scale information, significantly improving the model’s ability to detect small distant objects.
3. We applied channel pruning to the model, reducing its size, parameter count, and FLOPs by 50%, 55%, and 44%, respectively. The improved model was then trained and evaluated on three green fruit datasets. The enhanced model achieved accuracies of 94.5%, 84.4%, and 85.9% on the Korla Pear, Guava, and Green Apple datasets, respectively—improvements of 1.17%, 1.1%, and 1.77% over the baseline model. Additionally, the mAP@0.5 increased by 0.72%, 6.5%, and 0.9%, while the recall rate improved by 1.97%, 1.1%, and 0.49%, respectively.

The remaining sections of this paper are organized as follows: Section 2 describes the YOLOv8 network model, GreenFruitDetector architecture composition, and pruning scheme. Section 3 provides the details of the experiments, including experimental setup, dataset acquisition, evaluation metrics, furthermore, we illustrated and analysis our metric and visual result, ablation experiments we conducted. Finally, Section 4 presents a summary of the main work and achievements of the study.

## 2. Materials and methods

Our model builds upon enhancements made to YOLO v8. In this section, we first introduce the dataset acquisition process and outline the structure of YOLO v8. We then provide an overview of the GreenFruitDetector’s overall architecture. Following this, we detail two key improvements: the Multi-path Coordinate Attention Guided Deformer Convolution (MCAG-DC) and the Fusion-neck. Finally, we present our strategy for model pruning and compression.

### 2.1 Data acquisition

**Fig 1:**
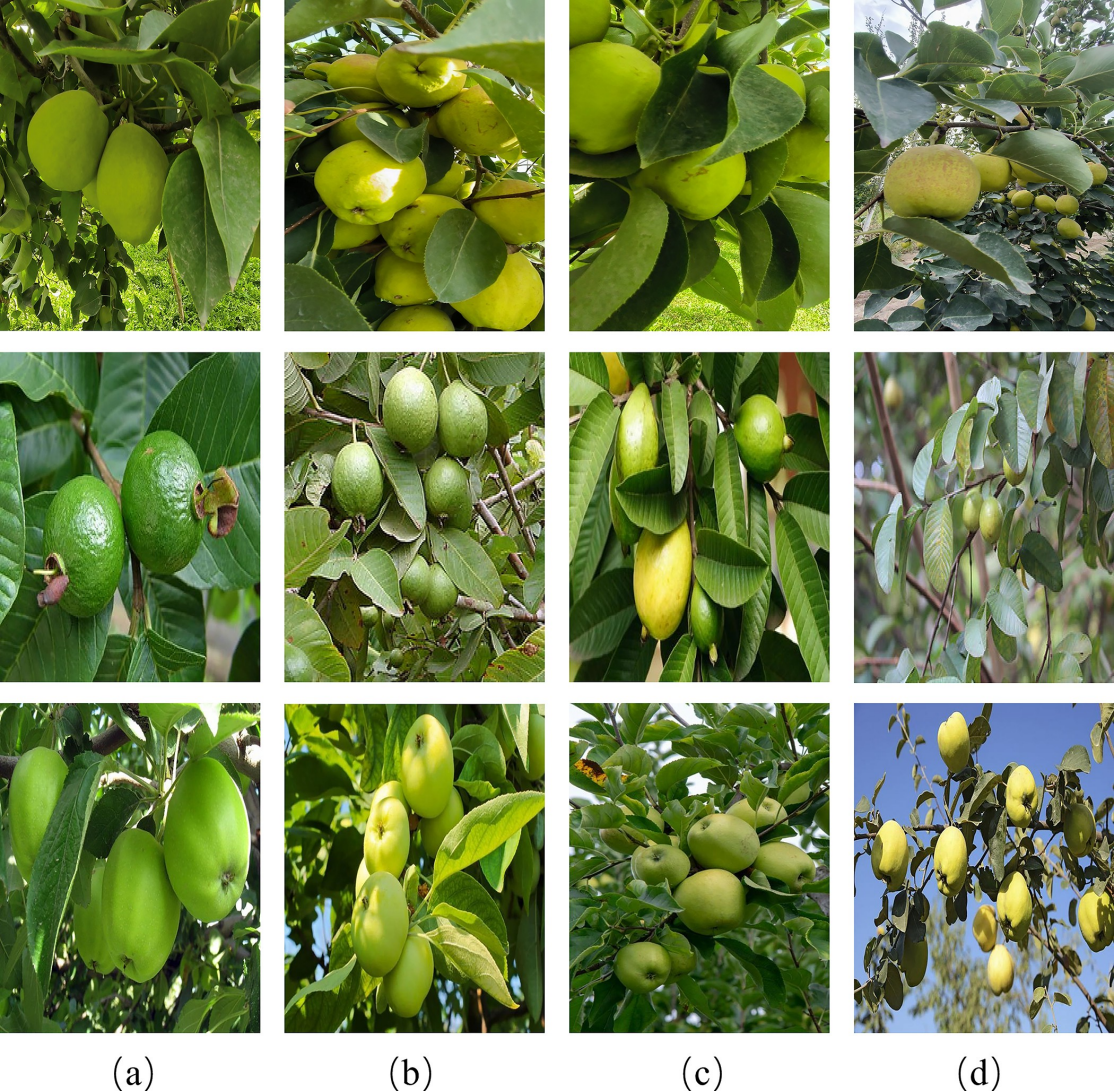
Top row: Korla fragrant pear dataset. Middle row: Guava dataset. Bottom row: Green Apple dataset. (a) Non-occluded target; (b) Fruit overlap; (c) leaves occlusion; (d) object in the distance. Reprinted from [guava Dataset] under a CC BY license, with permission from [Roboflow], original copyright [2024].

In this study, to demonstrate the effectiveness and generalization of our model, we collected three types of green fruits: Korla fragrant pear, Guava and green apple. Fig 1 displays the examples from three datasets under various complex scenarios.

Korla fragrant pear dataset: We selected Korla pears as the subject of our study, and the images were collected from a standardized Korla fragrant pear orchard, which covers an area of approximately 3.3 hectares. Within the orchard, the spacing between pear trees is about 3 meters, and the average tree height is about 3.5 meters. These parameters provide suitable spatial conditions for the operation of a pear-picking robot within the orchard. Using a Canon camera, we captured 1,201 images of Korla pears at various distances. To simulate the picking operation of a picking robot, images were collected from multiple angles, including scenes where pears were obscured by leaves, overlapping pears, pears with similar background colors, and pears in the distance. The captured images come in two resolution specifications: 1800 x 4000 pixels and 3072 x 1728 pixels, all stored in JPEG format. The dataset was strictly divided into training set (961 images), validation set (120 images), and test set (120 images) in a ratio of 8:1:1. We used the LabelImg software to annotate the Korla fragrant pears with minimum bounding rectangles.

Guava dataset [28]: A public dataset of Guava images provided by RoboFlow, which includes a total of 170 images. The images are annotated in YOLOv8 format, comprising 120 for training, 33 for validation, and 17 for testing. The following pre-processing steps were applied to each image: Auto-orientation of pixel data (with EXIF-orientation stripping), Resize to 640x640.

Green Apple Dataset: The dataset consists of 4,000 apple images acquired by filtering the MinneApple dataset [29] and web scraping, with resolutions of 1280x720 and 640x640. After further filtering, 2431 images that fit the criteria for green fruit detection in orchard environments were annotated. The apples were annotated with minimum bounding rectangles using LabelImg. These images were divided into a training set (1,942 images), a validation set (245 images), and a test set (244 images) to ensure the accuracy of model training and evaluation. Table 1 presents the total size and characteristics of the three datasets.

**Table 1:** Description and distribution of datasets.

Dataset Name	Total	Img Size	Training Set	Val/Test
**Korla fragrant pear**	1201	1800×4000, 3072×1728	961	120/120
**Guava**	170	640×640	120	33/17
**Green Apple**	2431	1280×720, 640×640	1942	245/244

### 2.2 YOLOv8 network architecture

**Fig 2:**
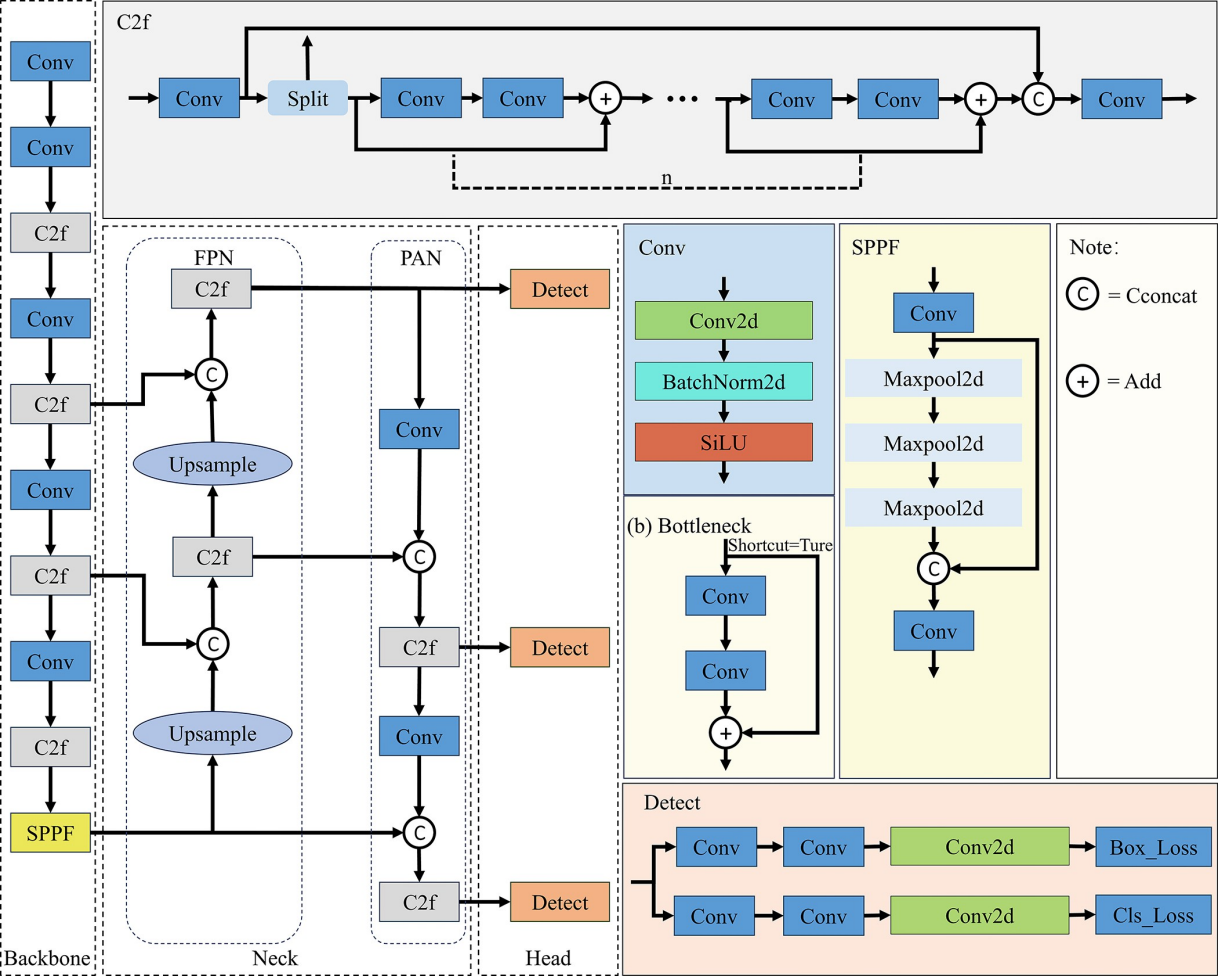
Architecture of original YOLOv8 network.

YOLOv8 is a significant technology in the field of real-time object detection, comprising three main components: Backbone, Neck, and Head, as illustrated in Fig 2. The Backbone, a convolutional neural network, is primarily responsible for extracting image features at various granularities, resulting in five feature maps of different scales. It consists of ConvBNSiLU (Conv) modules, CSPLayer_2Conv (C2f), and the spatial pyramid pooling-fast (SPPF) module. This integrated structure enables YOLOv8 to maintain its lightweight nature while capturing richer gradient flow information. The Neck section predominantly conducts feature fusion, by inputting the feature branches P3, P4, and P5 from the Backbone into the Path Aggregation Networks Feature Pyramid Networks [30, 31] (PAN-FPN) structure for multi-scale fusion. This architecture achieves effective detection of multi-scale targets through a bidirectional fusion Backbone network, which integrates both top-down and bottom-up approaches. The detection head is divided into two independent branches—one for object classification and the other for bounding box prediction. The bounding box prediction employs the Distribution Focal Loss (DFL) [32] and Complete Intersection over Union (CloU) techniques, enhancing detection precision and expediting model convergence.

### 2.3 GreenFruitDetector

The YOLOv8 model excels in many areas but struggles to overcome the challenges of detecting fruits with color similarity to leaves in orchard environments. We believe this is primarily due to two main shortcomings:

- Insufficient Feature Extraction: The current feature extraction network inadequately captures the diverse shapes and scales of fruits, particularly when obscured. This often results in missed detections or false positives.
- Inadequate Feature Fusion: The existing PAN and FPN structure does not effectively process large-scale feature maps, leading to the loss of crucial features and diminished detection quality. Additionally, for small objects at the pixel-level resolution, the fusion of the target layer P3 does not adequately meet the positional information requirements.

**Fig 3:**
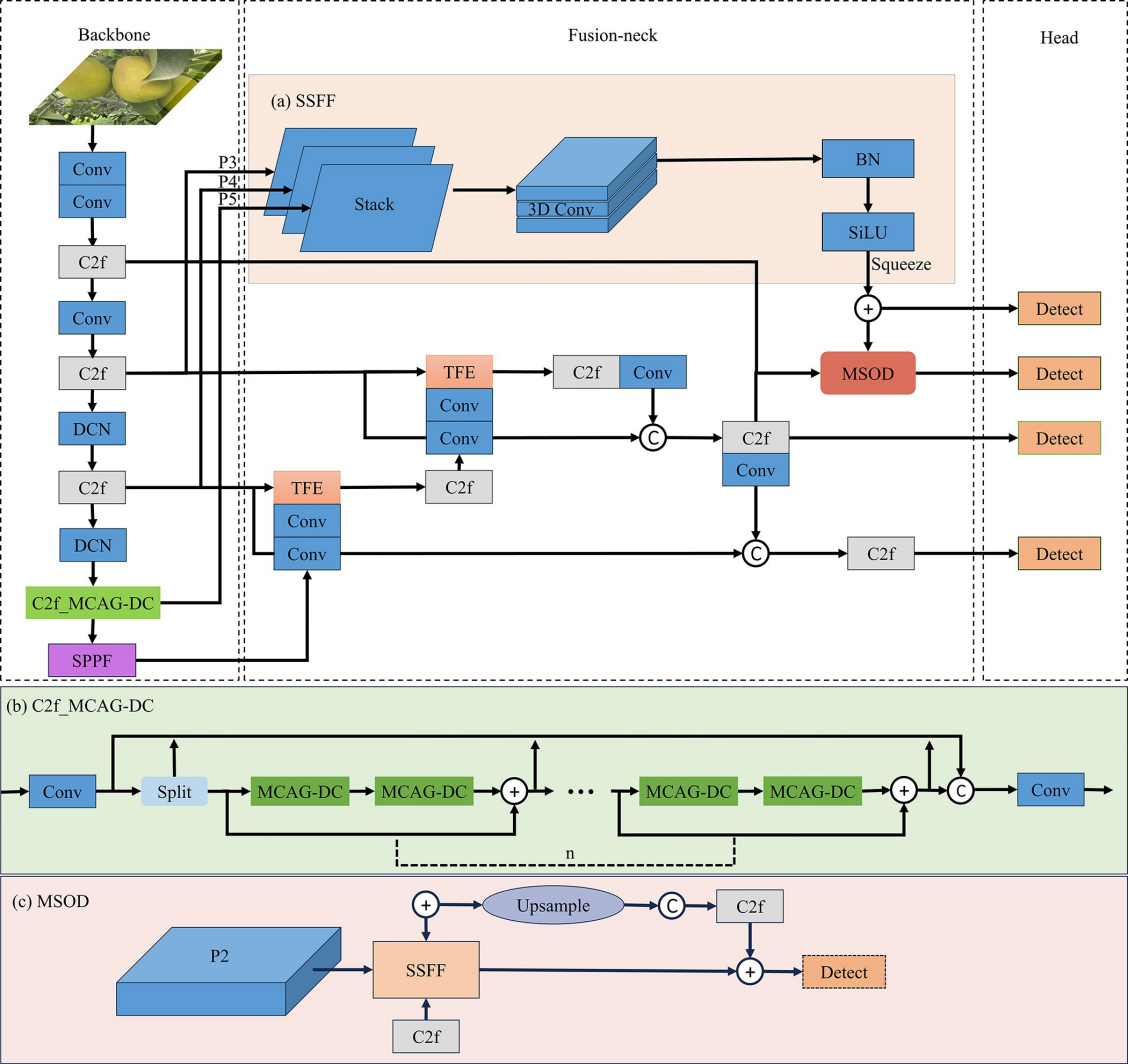
Architecture of GreenFruitDetector network. Model improvement: The Backbone utilizes the DCN and C2f_MCAG-DC module to enhance feature extraction capability; the Fusion-neck is composed of components (a)SSFF, (b)TPE and (c) MSOD.

Based on these, we devised a novel deformable convolution layer, called MCAG-DC, which utilized Multi-path Coordinate Attention guides computing the offset of deformable convolution to enhance the model extract geometric features. Additionally, a Fusion-neck structure is proposed in the Neck network. We introduce the Scale Sequence Feature Fusion Module (SSFF) [33] and Triple Feature Encoding Module (TFE) [33] to enhance multi-scale high-level semantic information. Furthermore, we devise a Multiscale Small Object Detector (MSOD) to augment the model’s detection capability for small objects in distant views. The overall architecture is shown in Fig 3.

**Fig 4:**
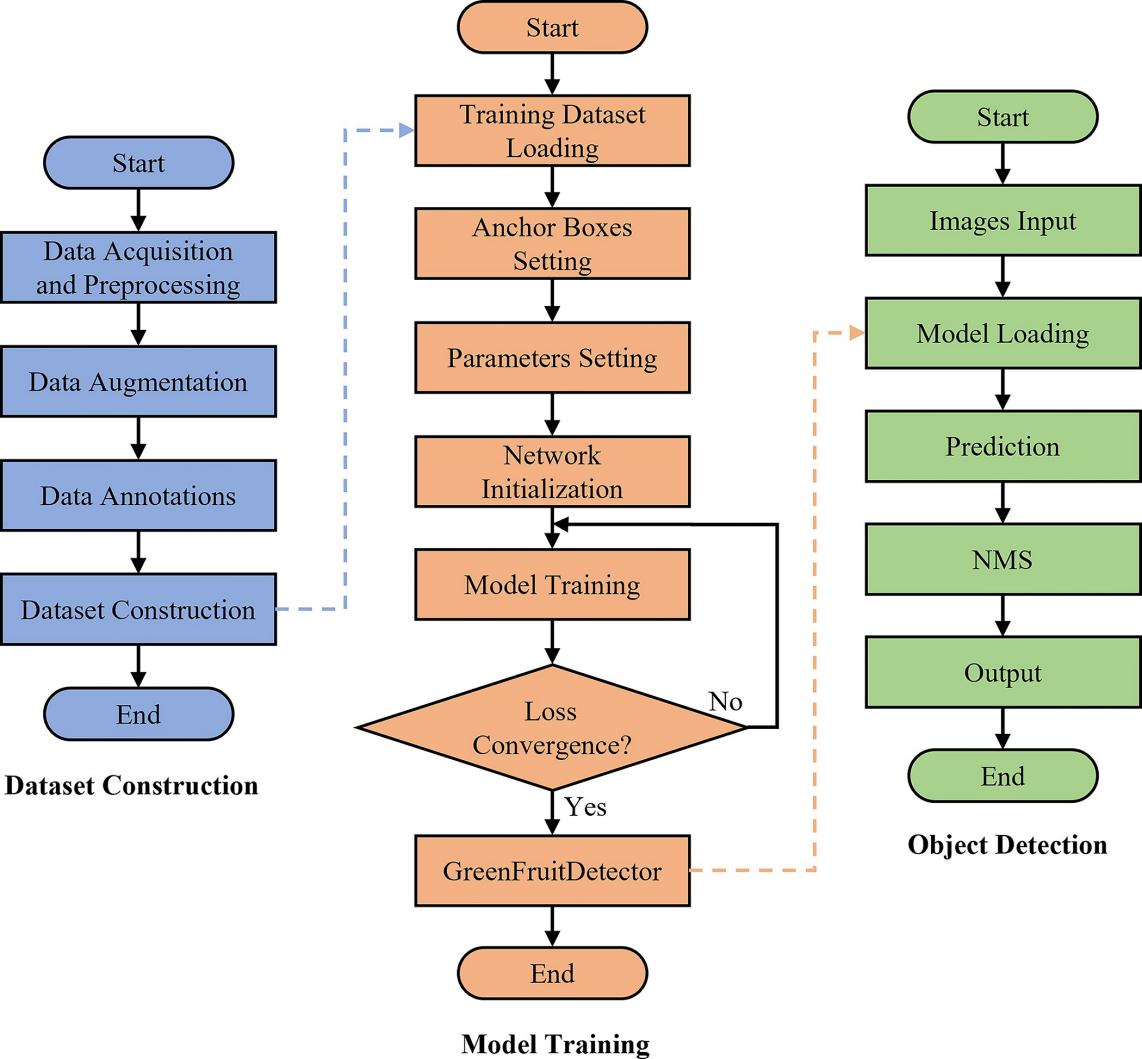
The flowchart of GreenFruitDetector.

Fig 4 illustrates the process of the GreenFruitDetector for object detection. The target detection pipeline comprises three key stages: dataset construction, model training, and inference. Initially, the acquired images undergo preprocessing and precise annotation using Labelimg. Following this, training parameters are set, and the model is loaded for training. DFL is used for bounding box refinement, while CIoU loss improves accuracy. The model weights are iteratively updated to converge the loss function, leading to the final GreenFruitDetector model, optimized for target detection.

#### 2.3.1 MCAG-DC: Multi-path Coordinate Attention Guided Deformable Convolution

**Fig 5:**
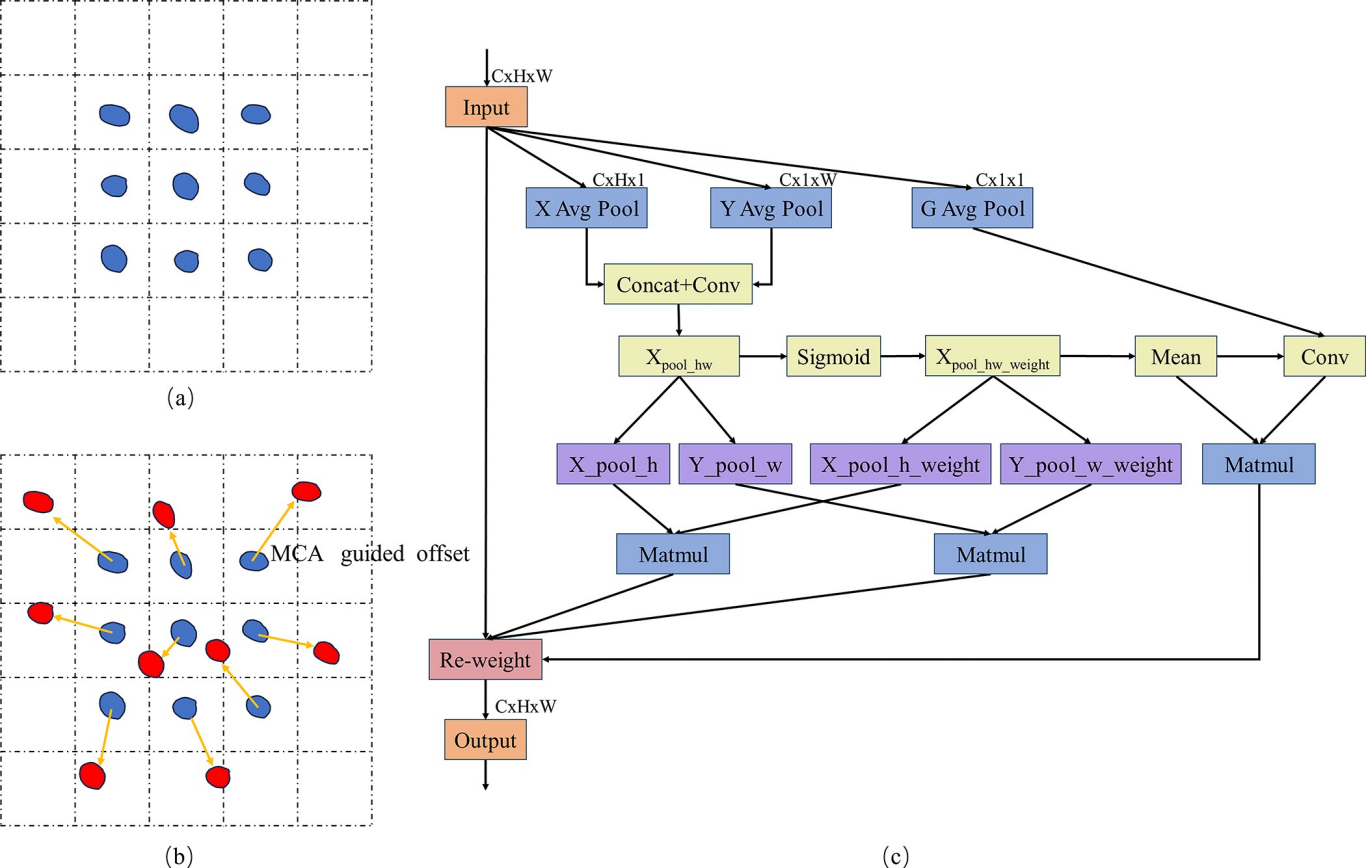
(a) Standard Convolution; (b) Deformable Convolution (Offset mask). (c) Structure Diagram of Multi-path Coordinate Attention (MCA) Mechanism, including height-direction pooling, width-direction pooling, and global pooling.

Standard convolution uses a fixed, uniform kernel that moves across the input feature map according to a set stride parameter, limiting its ability to capture irregular shapes and complex geometric deformations. In contrast, deformable convolution introduces offset parameters that allow the convolutional kernel to adjust its position on the feature map, enabling it to adapt to geometric deformations in the input image. Fig 5(A) and 5(B) illustrates the difference between standard convolution and deformable convolution. However, offset parameters rely on local features, this may lead to inaccurately calculate the offset parameters In cases of occlusion or noise. To address this issue, inspired by Coordinate Attention [34], We designed the Multi-path Coordinate Attention Guided Deformable Convolution module, which is guided by MCA to adjust the offsets of deformable convolution. The Structure Diagram of MCA is illustrated in Fig 5(C).

The MCA is a novel and efficient multi-scale attention module for cross-space learning. It employs a three-branch structure that enables the offsets of deformable convolutions to extend along both horizontal and vertical directions. Additionally, global pooling is integrated to focus attention on regions of interest while preserving information on each channel.

The calculation process of MCA(x) is as follows:

Assuming the input x∈ℜ^*C*×*H*×*W*^, calculate adaptive average pooling for both height and width direction separately, the third path calculate global average pooling.


$$x_{pool\_h}=AdaptiveAvgPool(x),$$ (1)

$$y_{pool\_w}=AdaptiveAvgPool(x),$$ (2)

$$x_{pool\_ch}=Conv(AdaptiveAvgPool(x))$$ (3)

Then, merge the height and width direction information and perform convolutional operation to obtain *x*_*pool*_*hw*_.


$$x_{pool\_hw}=Conv(Concat(x_{pool\_h},y_{pool\_w}))$$ (4)

Next, height-direction attention weights (*x*_*pool*_*h*_*weight*_) and width-direction attention weights ((*y*_*pool*_*w*_*weight*_), can be caculated by Eq 5.


$$x_{pool\_h\_weight},y_{pool\_w\_weight}=Split(Sigmoid(Conv(x_{pool\_hw})))$$ (5)

Eq (6) perform weighted operations on the *x*_*pool_h*_ and *y*_*pool_w*_.


$$x_{pool\_h}=Matmul(x_{pool\_h},x_{pool\_h\_weight})$$

$$y_{pool\_w}=Matmul(y_{pool\_w},y_{pool\_w\_weight})$$ (6)

Similarly, perform weighted operations on the global average pooling as shown in Eq (7).


$$x_{pool\_ch}=x_{pool\_ch}\cdot Mean(sigmoid(x_{pool\_hw\_weight}))$$ (7)

After performing the preceding calculations, we derive the final output result for MCA by Eq (8).


$$MCA(x)=x\cdot Sigmoid(x_{poo\_h})\cdot Sigmoid(y_{pool\_w})\cdot Sigmoid(x_{pool\_ch})$$ (8)

**Fig 6:**
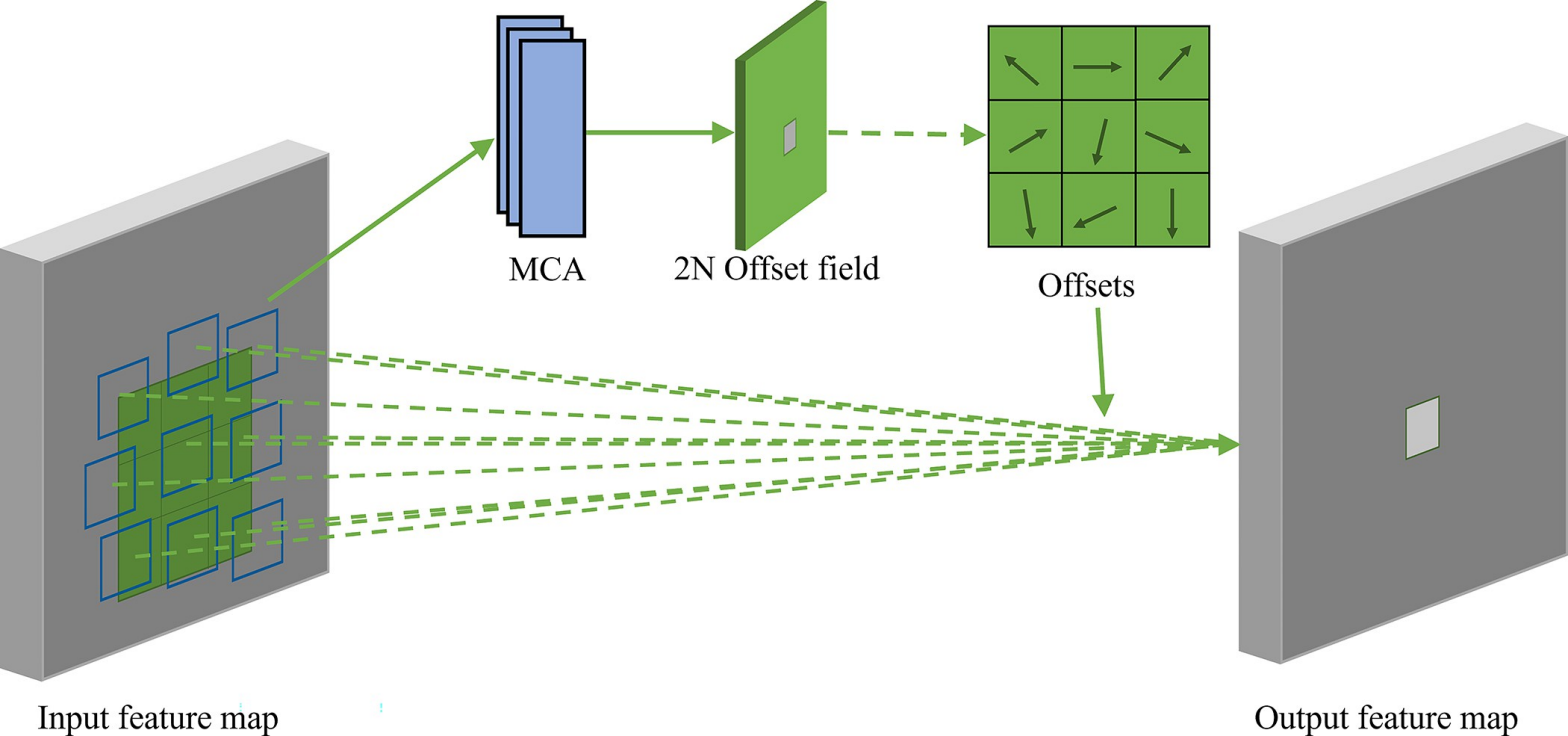
Structure of MCAG-DC.

The workings of MCAG-DC are depicted in Fig 6. Firstly, the input image is convolved with traditional convolutional kernels to extract initial feature maps. Subsequently, these feature maps are fed into a convolutional layer guided by the MCA which is responsible for computing the offset of each convolutional kernel element in both the x and y directions, forming 2N output channels, where N is the number of channels of the convolutional kernel. The obtained offsets can dynamically adjust their shapes and positions according to the content of the input image. The convolution calculation formula is shown in Eq 9.

The computation process of MCAG-DC can be divided into two steps: (1) Utilizing a regular grid for sampling on the input feature map. (2) Conducting weighted operations on the values obtained at each sampling point.

For example, R = {(-1, -1), (-1, 0), …, (0, 1), (1, 1)} defines a 3 × 3 convolutional kernel of dilation 1. Where R is a regular grid for sampling on the input feature map.

The mapping relationship of the feature map can be articulated through the use of Eq(9).


$$y(p_{0})=\sum_{p_{n}\in R}w(p_{n})\cdot x(p_{0}+p_{n}+\Delta p_{n})$$ (9)

Where *w*(*p*_*n*_) represents the weight at position *p*_*n*_. *p*_*o*_ represents the original position of the feature map, while *p*_*n*_ signifies the positions listed in the sampling points. Δ*p*_*n*_ represents the offset of the deformable convolution, which is caculated in Eq (10).


$$\Delta p_{n}=MCA(x)$$ (10)

We replaced all convolutional layers in the C2f module at the 9th layer of the Backbone with our MCAG-DC, forming the C2f_MCAG-DC module, as illustrated in Fig 3(B).

#### 2.3.2 Fusion-neck

In the original YOLOv8 setup, there are three detection heads corresponding to feature map sizes of 80×80, 40×40, and 20×20 in the Neck, responsible for detecting small, medium, and large-scale targets, respectively. However, the detection capability of YOLOv8’s detection heads for small targets remains limited, and the richer information about small targets in the shallow feature maps of the Backbone network and Neck end has not been fully utilized. Considering this, we designed the Fusion-neck, which includes the SSFF module and TFE module in the Neck to enhance the model’s ability to fuse multi-scale features. Additionally, it introduces a new detection head, MSOD, which fuses multi-scale features to improve the network’s perception of small targets.

In the SSFF module, the p3, p4, and p5 (Fig 3 Backbone) feature maps are upsampled and normalized to the same size, then stacked together as the input for 3D convolution to combine multi-scale features. The subsequent processing within the SSFF module, applied to the scaled images, is detailed below:

$$F_{0}(w,h)=G_{0}(w,h)\cdot f(w,h)$$ (11)

$$G_{0}(w,h)=\frac{1}{2\pi \sigma^{2}}e^{-(w^{2}+h^{2})/2\sigma^{2}}$$ (12)

where *f*(*w*, *h*) represents a 2D input image with width w and height h. *F*_0_(*w*, *h*) is generated by smoothing with a series of convolutions using a 2D Gaussian filter *G*_0_(*w*, *h*). σ serves as the scaling parameter for the standard deviation of the 2D Gaussian filter used in the convolution. The structure diagram of the SSFF module is shown in Fig 3(A).

**Fig 7:**
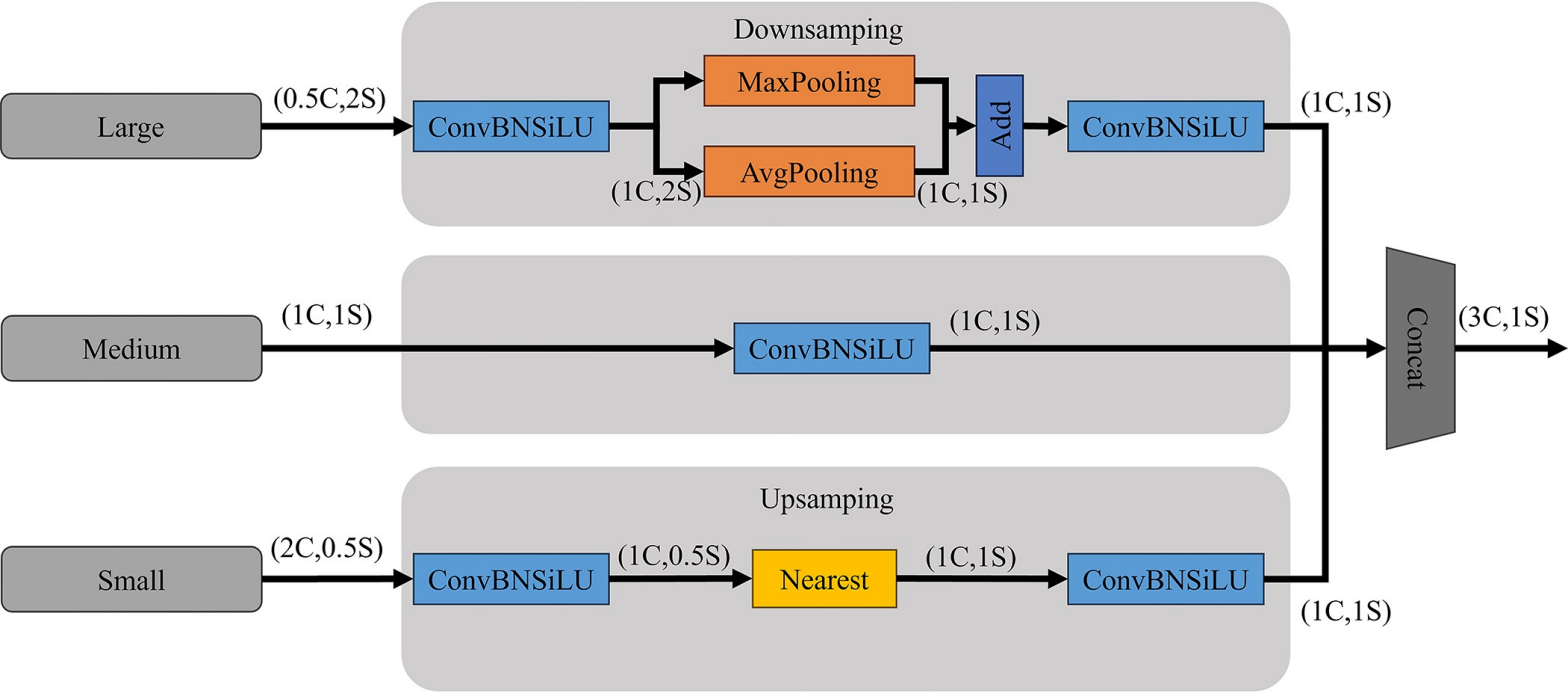
Structure diagram of the TFE module.

The TFE module is an innovative feature fusion mechanism designed to handle input feature maps of different sizes. It concatenates features of large, medium, and small sizes in the spatial dimension to capture detailed information about small targets. The module then performs ConBNSiLU (including convolution, batch normalization, and SiLU activation function operations) on these concatenated features. Subsequently, upsampling and downsampling techniques are employed to adjust the spatial dimensions of the feature maps, ensuring consistency in size and enhancing the detection capability in scenarios where green fruits are densely clustered. The structural diagram is depicted in Fig 7.

#### 2.3.3 Multiscale Small Object Detector (MSOD)

With the increase in layers, small targets and detailed information are prone to being overlooked. Taking this into consideration, we have further designed a detection head named MSOD, which integrates the SSFF module design to fuse different scale feature maps to enrich semantic information.

The MSOD combines the features maps of the 3rd, 20th, and 25th layers, forming a deep semantic feature layer containing targets with more detailed low-level information and even smaller targets. This further enhances the network’s feature fusion capability, enabling better preservation of relevant feature information for small targets at a large scale of 160×160 and increasing the model’s resolution. The introduction of additional decoupled heads extends the detection range for green fruits, allowing the model to detect targets with more detailed low-level information and even smaller targets. The structure of the MSOD is shown in Fig 3(C).

### 2.4 Model pruning and compression

**Fig 8:**
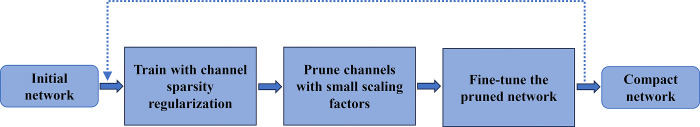
Pruning flowchart.

The channel pruning module consists of three steps: sparse training, channel pruning, and fine-tuning training. The flowchart is shown in Fig 8.

#### 2.4.1 Sparse training

The purpose of sparse training is to differentiate between important and non-important channels, enabling subsequent pruning of non-important channels. Batch normalization (BN) layer are utilized to perform sparse training on the network:

$$\hat{z}=\frac{z_{in}-\mu_{B}}{\sqrt{\sigma_{B}^{2}+\varepsilon }},z_{out}=\gamma \hat{Z}+\beta ,$$ (13)

where $z_{in}$ and $z_{out}$ are the input and output of the BN layer, respectively. B represents each batch during training; *μ*_*B*_ and *σ*_*B*_ represent the mean and standard deviation of each batch B; *γ* and *β* are the scaling and shifting factors.

The *γ* parameter of the BN layer after each convolutional layer in the network is used as a scaling factor to evaluate the importance of channels, thus obtaining the overall loss function for channel pruning:

$$L=\sum_{\left(x,y\right)}l(f(x,W),y)+\lambda \sum_{\gamma \in \Gamma }g(\gamma )$$ (14)

Where *x* and *y* denote the training input and output respectively, and *W* represents the training parameters. The second term on the right-hand side represents the L1 regularization constraint using scaling factors, where *λ* is the sparsity training ratio factor, Γ is the set of all scaling factors in the BN layer, and *g*(*γ*) represents the penalty term for scaling factors.

#### 2.4.2 Channel pruning and fine-tuning

After sparsity training, the scaling factors of the BN layer are ranked based on their absolute values. Subsequently, a pruning ratio is determined to specify the proportion of channels to be removed. Clearly, the higher the pruning ratio, the smaller the resulting model, but this may lead to a decrease in detection accuracy. Following model pruning, there is typically some decrease in detection accuracy. Therefore, iterative training of the network model is necessary to fine-tune the model weights and restore accuracy.

## 3. Results and analysis

### 3.1 Experimental environment

The experiment was conducted on the CentOS 7 operating system, utilizing an Intel Xeon Silver 4110 CPU and an NVIDIA Tesla V100 GPU. The programming language Python 3.8 was employed, with PyTorch version 1.13.1 serving as the designated deep learning framework. Throughout the training period, optimization was achieved through stochastic gradient descent (SGD), employing an initial learning rate of 0.01, a momentum factor of 0.937, and a weight decay rate of 0.0005. The input images were standardized to a size of 640×640, and the batch size was set to 32. The training lasted for more than 200 epochs, with early stopping set to 50 epochs. Mosaic data augmentation and mixed training methods were enabled during the training process. The experimental parameters are shown in Table 2.

**Table 2:** Experimental parameters.

Attribute	Value
**Input Image Size**	[640,640]
**Initial Learning Rate**	0.01
**Optimizer**	SGD
**Momentum Factor**	0.937
**Batch Size**	32
**Epoch**	200
**Early Stopping**	50
**Weight Decay**	0.0005
**Data Augmentation**	Mosaic data augmentation, mixed training methods

### 3.2 Evaluation indicators of model

This experiment utilizes the following metrics to evaluate the performance of object detection models: Precision, Recall, and mean average precision (mAP) at IoU thresholds of 0.5 (mAP@0.5) as measures of detection accuracy. Additionally, network parameter counts (Params), floating-point operations (FLOPs), and inference time are considered indicative measures of both efficiency and real-time performance. The size of the model weight (model size) is utilized to assess the model’s suitability for deployment on edge devices. The formulas for Precision, Recall, and mAP are shown in Eqs (15), (16), and (17), respectively.


$$Precision=\frac{TP}{TP+FP}$$ (15)

$$Recall=\frac{TP}{TP+FN}$$ (16)

$$mAP=\frac{1}{N}\sum_{i=1}^{N}AP_{i}$$ (17)

Where TP denotes the count of true positive predictions. FN represents the count of negative samples correctly predicted as negative. FP represents the count of negative samples incorrectly predicted as positive, and N is the total number of categories.

### 3.3 Comparative experiments on the detection of different models

To demonstrate the effectiveness of our improved model for smart harvesting detection, we conducted comparative experiments against mainstream object detection algorithms, including YOLOv3-tiny, YOLOv5s, YOLOv7-tiny, YOLOv8n, and RT-DETR [35], across three datasets.

As shown in Tables 3–5 in terms of the number of parameters and model size metrics, GreenFruitDetector has the lowest number of parameters, which is 55.2% less than the YOLOv8n baseline model, and the model size is only half of the baseline. Additionally, our model has the lowest FLOPs, being only one-tenth of RT-DETR and one-third of the YOLO series models. These indicate that our model is more lightweight. For inference speed, the GreenFruitDetector takes 1.3 ms, 1.0 ms, and 1.6 ms, respectively, on the Fragrant Pear, Guava, and Green Apple datasets. These times are significantly lower compared to other detection models. Without a doubt our model has the lowest complexity while being more efficient in storage and transmission and is suitable for resource-constrained environments. Meanwhile, GreenFruitDetector still performs impressively in Precision, mAP@0.5, and Recall metrics. Compared to the advanced YOLOv8n, the Precision on the three datasets increased by 1.17%, 3.81%, and 1.77% respectively. The mAP@0.5 also improved by 0.72%, 6.5%, and 0.9% respectively. Recall rate increased by 1.97%, 1.1%, and 0.49% respectively. Although the GreenFruitDetector demonstrates slightly lower precision on the Guava and Apple datasets, its parameter count and FLOPs are significantly lower than those of RT-DETR.

**Table 3:** Comparison of algorithms on Korla fragrant pear. (↑ means higher is better, ↓ means lower is better).

Algorithm	Params (M) ↓	Precision (%)	mAP@0.5 (%)↑	Recall(%)	FLOPs (G)↓	Model Size(MB) ↓	Inference (ms) ↓
**Yolov3-tiny**	8.65	90.1	91.2	83.1	13.0	17.4	3.1
**Yolov5s**	7.01	91.8	95.6	88.9	15.8	14.3	4.7
**Yolov7-tiny**	6.01	88.9	95.7	92.6	13.2	12.2	3.1
**Yolov8n**	3.20	93.4	96.3	91.2	8.1	6.0	1.0
**RT-DETR**	19.8	94.0	96.1	90.2	56.9	40.4	7.9
**GreenFruitDetector**	1.34	94.5	97.0	93.0	4.5	3.0	1.3

**Table 4:** Comparison of algorithms on Guava. (↑ means higher is better, ↓ means lower is better).

Algorithm	Params (M) ↓	Precision (%)	mAP@0.5 (%)↑	Recall(%)	FLOPs (G)↓	Model Size(MB) ↓	Inference (ms) ↓
**Yolov3-tiny**	8.65	77.3	76.4	72.0	13.0	17.4	3.7
**·Yolov5s**	7.01	81.4	74.9	70.3	15.8	14.3	3.5
**Yolov7-tiny**	6.01	78.1	69.1	69.6	13.2	12.2	2.8
**Yolov8n**	3.20	81.3	78.0	72.3	8.1	6.0	0.8
**RT-DETR**	19.8	84.6	75.5	68.2	56.9	40.4	9.9
**GreenFruitDetector**	1.34	84.4	83.1	73.1	4.5	3.0	1.0

**Table 5:** Comparison of algorithms on Green Apple. (↑ means higher is better, ↓ means lower is better).

Algorithm	Params (M) ↓	Precision (%)	mAP@0.5 (%)↑	Recall(%)	FLOPs (G)↓	Model Size(MB) ↓	Inference (ms) ↓
**Yolov3-tiny**	8.65	83.1	84.5	76.9	13.0	17.4	4.0
**Yolov5s**	7.01	85.5	87.7	80.2	15.8	14.3	3.9
**Yolov7-tiny**	6.01	83.6	87.0	79.7	13.2	12.2	4.2
**Yolov8n**	3.20	84.4	88.3	80.7	8.1	6.0	1.7
**RT-DETR**	19.8	86.0	89.3	79.5	56.9	40.4	8.0
**GreenFruitDetector**	1.34	85.9	89.1	81.1	4.5	3.0	1.6

**Fig 9:**
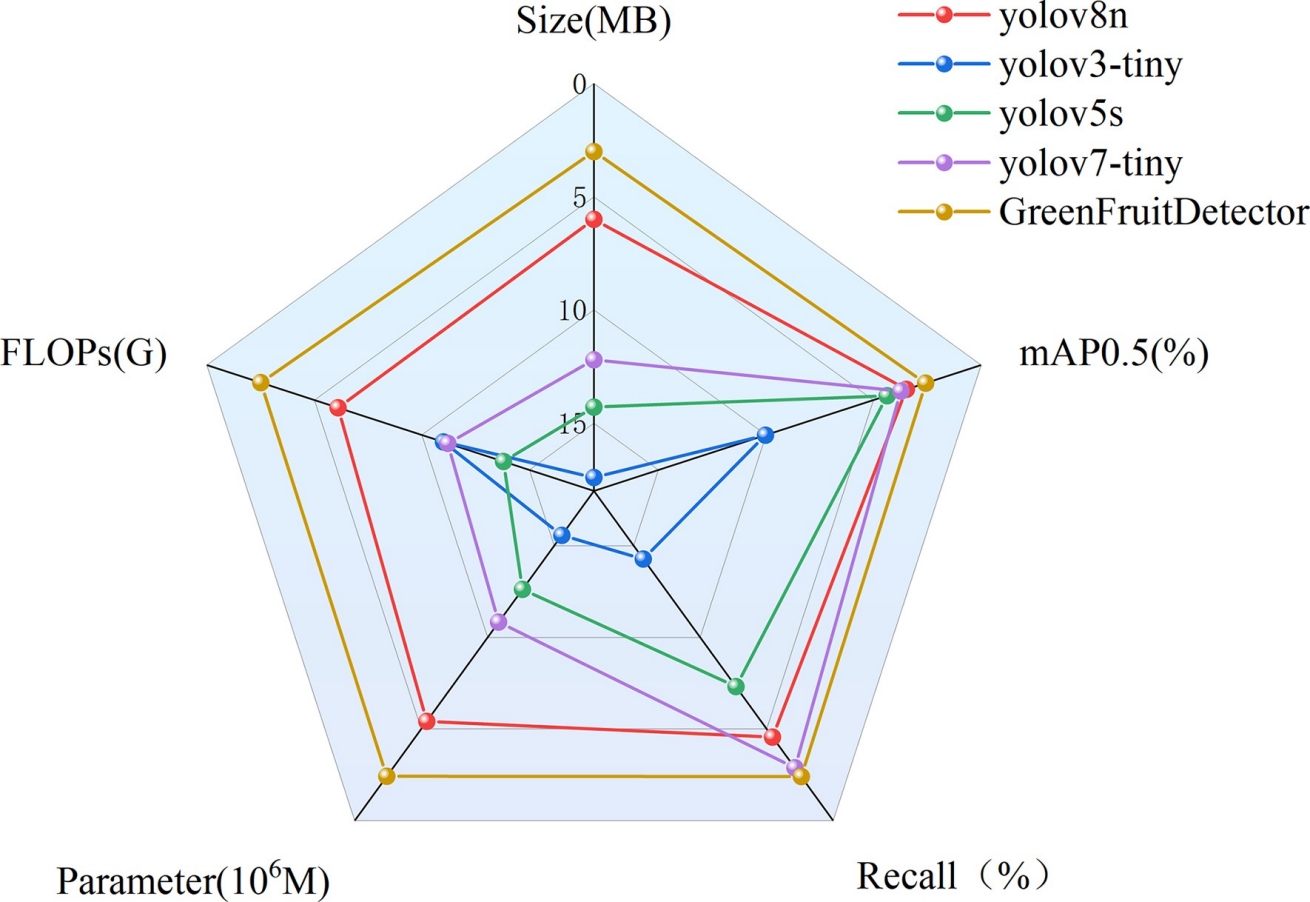
The Radar chart.

In summary, GreenFruitDetector achieves the best balance in detection accuracy, model size, and inference speed, meeting the requirements of smart harvesting detection tasks. In order to present the performance superiority of GreenFruitDetector over YOLO series models, Radar chart, shown in Fig 9, further visually illustrates the significant advantages of GreenFruitDetector in all aspects over other advanced detectors on the Korla fragrant detection dataset.

### 3.4 Comparison of improvement methods and results

Next, we analyze the impact of each module in the Backbone and Neck on the performance of the model separately. Our experiments are all conducted on Korla fragrant pear Dataset.

#### 3.4.1 Backbone improvement experiment

In the Darknet-53 backbone, there are multiple convolutional layers. To investigate the impact of using DCN to replace these layers on model performance, we designed four different structures and conducted experimental analysis.

The first one only utilizes DCN to replace the Conv layer in the Backbone (as shown in Fig 2). The second one only uses DCN to replace the convolutional layer in C2f. The third one only utilizes MCAG-DC to replace all convolutional layers in C2f. The fourth one is our Backbone, with DCN replacing Conv and with MCAG-DC replacing all convolutional layers in the fourth C2f. The experimental results are shown in Table 6.

**Table 6:** Comparison of model performance after Backbone improvement. (↑ means higher is better, ↓ means lower is better).

No.	Backbone	Params (M) ↓	Precision (%)	mAP@0.5 (%)↑	Recall(%)	FLOPs (G)↓	Model Size(MB) ↓	Inference (ms) ↓
**0**	**YOLOv8**	3.20	93.4	96.3	91.2	8.1	6.0	1.0
**1**	**Conv→DCN**	3.06	93.7	96.2	90.9	7.6	6.4	1.9
**2**	**C2f →C2f_DCN**	3.08	94.0	96.4	91.0	7.9	6.4	2.3
**3**	**C2f→C2f_MCAG-DC**	3.11	94.2	96.6	92.2	7.9	6.5	2.5
**4**	**Our Backbone**	3.08	95.0	96.7	92.9	7.6	6.4	2.0

It can be observed that after using DCN, the model’s Precision slightly increases, while Recall decreases. This indicates that although DCN optimizes the fine-grained recognition of targets, simply replacing standard convolutions does not effectively utilize its spatial detail capturing capabilities. To further enhance the Backbone feature extraction capability, we combine DCN with the C2f module, resulting in a 0.64% increase in Precision, indicating that incorporating deformable convolutions into the C2f module can expand the receptive field and improve model adaptability. Subsequently, by improving the C2f module with the MCAG-DC, the model’s performance further improves, with Precision, mAP@0.5 and Recall increasing by 0.85%, 0.3%, and 1%, respectively. Through our experiments, we found that fully replacing the layers with DCN or MCAG-DC resulted in an increase in accuracy, but also increased the model size. After multiple adjustments, we replaced the 4th and 5th standard convolutional layers in the backbone with DCN and enhanced the final C2f module in the backbone with the C2f_MCAG-DC structure. Its mAP@0.5 increases by 0.4%, Precision increases by 1.71%, and Recall also increases by 1.86%, all showing the best performance. This indicates that the Darknet-53 combined with the DCN and C2f_MCAG-DC structure can effectively handle targets in different complex situations and has better scale adaptability.

**Fig 10:**
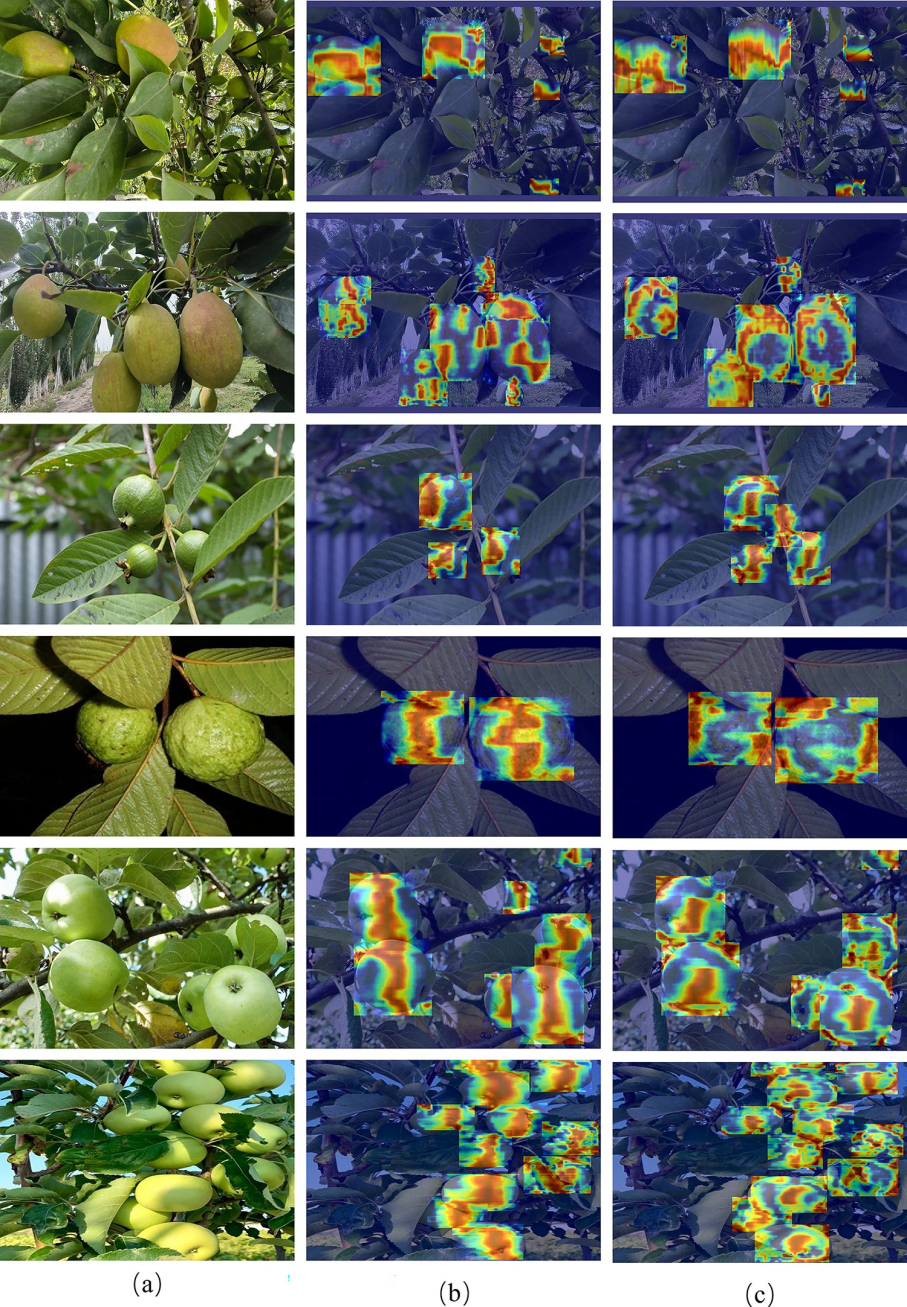
Comparison of heat maps for Korla fragrant pear dataset (Row 1–2), Guava dataset (Row 3–4) and Green Apple dataset (Row 5–6). (a) Original image. (b) yolov8n. (c) Backbone improved. Reprinted from [guava Dataset] under a CC BY license, with permission from [Roboflow], original copyright [2024].

To further demonstrate the effectiveness of our design, we compare the YOLOv8 Backbone with our improved Backbone and visualize their heatmaps. Fig 10 showcases six examples selected from the three datasets. It can be observed that our improved Backbone structure can effectively extract the edge feature information of occluded pears, which helps to focus attention on the edges of the pears, making it easier to distinguish fruits from the background and significantly enhancing the perception of occluded objects.

#### 3.4.2 Neck improvement experiment

Next, we will validate the effectiveness of the Fusion-neck. The first variant is the original baseline structure, the second one involves introducing SSFF and TPE into the neck part of the model. The third variant is YOLOv8-P2, a variation of YOLOv8 aimed at enhancing the detection of small objects. The fourth variant incorporates SSFF, TPE, and MSOD into the neck design structure, referred to as Fusion-neck. The experimental results are shown in Table 7.

**Table 7:** Comparison of model performance after Neck improvement. (↑ means higher is better, ↓ means lower is better).

No.	Model	Params (M) ↓	Precision (%)	mAP@0.5 (%)↑	Recall(%)	FLOPs (G)↓	Model Size(MB)↓	Inference (ms) ↓
**0**	**Yolov8**	3.20	93.4	96.3	91.2	8.1	6.0	1.0
**1**	**Yolov8+SSFF+TPE**	3.04	93.9	96.5	91.2	8.5	6.3	1.3
**2**	**Yolov8+P2**	2.92	93.7	95.9	91.9	12.2	6.2	1.5
**3**	**Fusion-neck**	2.48	94.6	96.6	93.2	12.0	5.3	1.3

When the Yolov8+SSFF+TPE framework is used for the Neck, experimental results show that there is a 0.5% improvement in Precision, with parameters remaining essentially unchanged and a slight increase in FLOPs. However, when we adopt the Yolov8+P2 small object detection layer, map@0.5 decreases while Precision increases by 0.3%, but there is a significant increase in FLOPs. In order to both ensure high accuracy and further improve the detection of distant small objects, we designed the Fusion-neck structure. Experimental results show that the parameter count is reduced by 17%, and the model size only requires 5.3M. At the same time, mAP@0.5, Precision, and Recall are improved by 0.3%, 1.28%, and 2.19% respectively compared to yolov8, meeting the accuracy requirements for the Korla pear detection in orchard environments.

### 3.5 Ablation experiment

To validate the effectiveness and significance of the improved model enhancement strategy, this paper conducted ablation experiments, and the experimental results are shown in Table 8.

**Table 8:** Ablation experiments. (↑ means higher is better, ↓ means lower is better).

DCN+C2f_MCAG-DC	Fusion-neck	Params (M) ↓	Precision (%)	mAP@0.5 (%)↑	Recall(%)	FLOPs (G)↓	Model Size(MB)↓	Inference (ms) ↓
*×*	*×*	3.20	93.4	96.3	91.2	8.1	6.0	1.0
*√*	*×*	3.08	95.0	96.7	92.9	7.6	6.4	2.0
*×*	*√*	2.48	94.6	96.6	93.2	12.0	5.3	1.3
*√*	*√*	2.57	95.5	97.3	94.3	11.5	5.5	1.6

**Fig 11:**
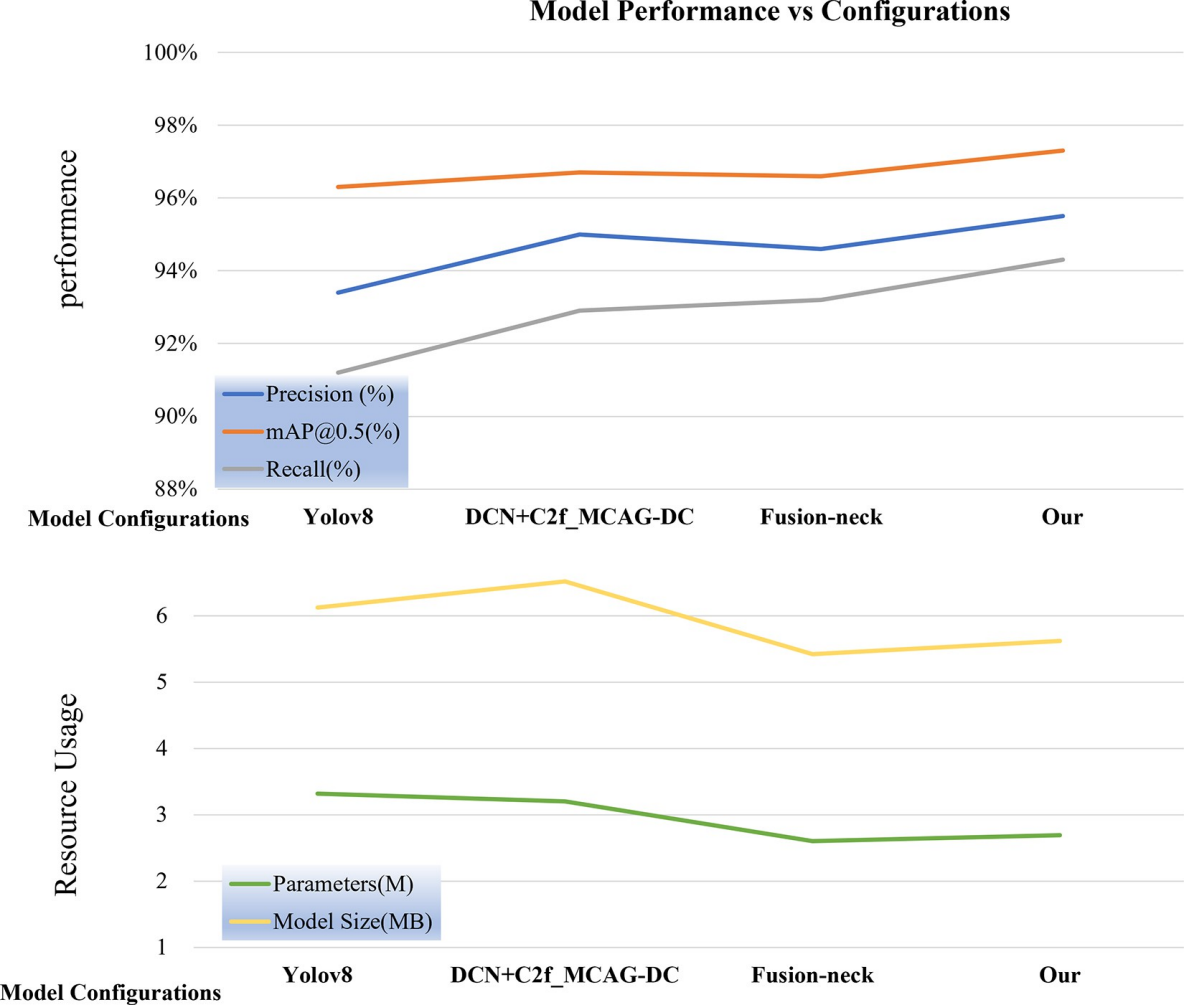
Comparison of model performance resource usage.

It can be observed that by incorporating the DCN and C2f_MCAG-DC combination structure into the Backbone network, the detection accuracy showed improved performance. The Map@0.5 increased by 0.4%, Precision increased by 1.7%, and Recall increased by 1.86%. At the feature fusion stage, adopting the Fusion-neck design integrated with MSOD not only results in fewer parameters and significant accuracy improvements but also achieves faster inference times, requiring only 1.3 ms. The model achieved performances of 96.6% for Map@0.5 and 94.6% for Precision, with a 17% reduction in parameters compared to yolov8n. When employing advanced Backbone structures and lightweight feature fusion modules simultaneously, the use of deformable convolutions enables the model to better capture features in complex scenes. During feature fusion in the Fusion-neck, the model’s detection capability for targets of different sizes and shapes is enhanced, further improving precision. At this point, the model’s Precision, Map@0.5, and Recall increased by 2.24%, 1.03%, and 3.39% respectively compared to yolov8n, with a 14.4% reduction in parameters and an 8.33% reduction in model size, significantly reducing the utilization of computational resources. Fig 11 illustrates the performance and resource utilization comparison of the experimental results. By comparing the points on the far left and far right columns, the importance of improvement strategies on detection performance can be observed more clearly, validating the effectiveness of each strategy.

### 3.6 Pruning experiment

**Fig 12:**
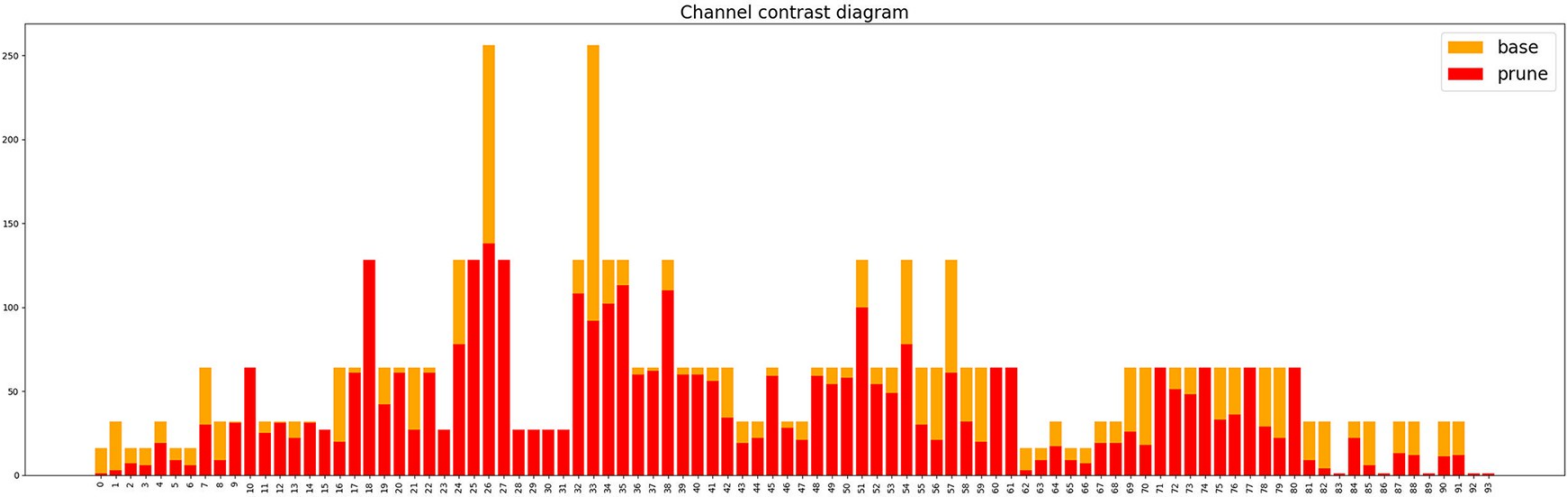
The channel changes of each layer base and after prune.

We further experimented with the setting of pruning rates. Setting an appropriate pruning rate can optimize the overall performance in terms of accuracy and model size. To ensure the integrity of the network structure, at least one channel must be retained. Therefore, in this study, pruning ratios of 1.0x, 1.5x, 2.0x, 2.5x, 3.0x, and 3.5x are set. After pruning, finetuning was conducted to restore the model’s detection accuracy, and the pruning ratio with the best overall performance is selected as the final model. Table 9 presents the optimization results of different pruning ratios on network performance and Fig 12 demonstrates the variation in the number of model channels before and after sparse training.

**Table 9:** Improvement results of network performance by different pruning ratios. (↑ means higher is better, ↓ means lower is better).

Pruning ratio	Params (M) ↓	Precision (%)	mAP@0.5 (%)↑	Recall(%)	FLOPs (G)↓	Model Size(MB)↓	Inference (ms) ↓
**1.0x**	2.57	95.5	97.3	94.3	11.5	5.5	1.6
**1.5x**	2.08	94.3	96.5	91.6	7.6	4.5	1.6
**2.0x**	1.61	93.0	96.4	92.2	5.7	3.6	1.5
**2.5x**	1.34	94.5	97.0	93.0	4.5	3.0	1.3
**3.0x**	1.29	92.9	96.5	90.5	3.8	2.9	1.8
**3.5x**	1.16	92.4	95.7	91.1	3.2	2.7	1.9

According to Table 9, there is a certain degree of fluctuation in the model’s mAP@0.5 and Precision as the pruning ratio increases. From a pruning ratio of 1.0x to 2.0x, mAP@0.5 slightly decreases, reaches its peak at 2.5x, then decreases slightly. Meanwhile, the performance of Precision gradually decreases between 1.0x and 2.0x, then slightly recovers at 2.5x before declining again. This performance fluctuation may be attributed to the loss of some important feature channels during the pruning process, thereby affecting the model’s performance on certain samples. In such cases, a pruning ratio of 2.5x may be a relatively ideal choice.

### 3.7 Visualization results

**Fig 13:**
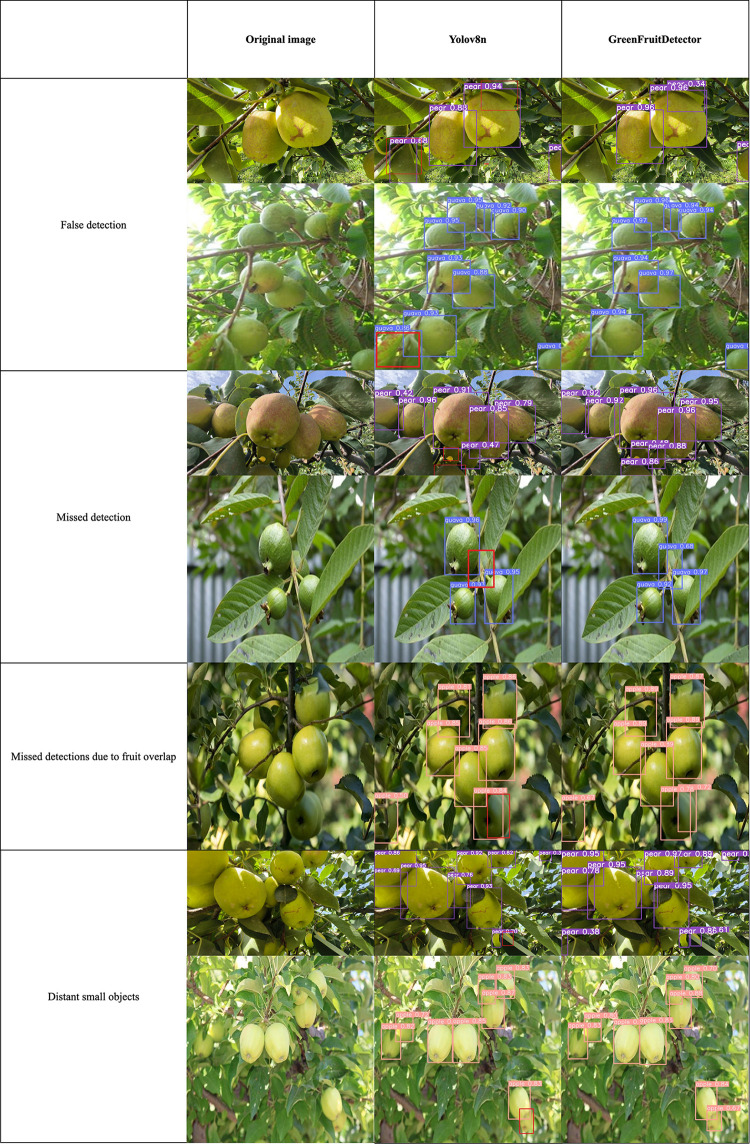
Comparison of test result between YOLOv8n and GreenFruitDetector. (Red boxes represent missed detections). Reprinted from [guava Dataset] under a CC BY license, with permission from [Roboflow], original copyright [2024].

From the experimental results, it is apparent that the proposed model in this research outperforms YOLO v8n and other mainstream object detection models in green fruit detection. Fig 13 presents a comparison of the detection performance between the proposed model and YOLO v8n on three datasets.

The comparative analysis in the figures above clearly demonstrates that the GreenFruitDetector proposed in this study outperforms the baseline model in detecting green fruits, especially in cases of fruit overlap and leaf occlusion, with an associated increase in confidence scores. In the first set of figures, when pears are in the background and partially occluded, the YOLOv8n model tends to overlook certain targets, leading to missed detections. In contrast, the GreenFruitDetector, by leveraging deformable convolutions, enhances semantic contextual information, thereby reducing the omission of heavily occluded targets. In the second and third sets of figures, the YOLOv8n model shows instances of false positives, whereas the GreenFruitDetector, with its multi-scale feature fusion design, improves sensitivity to critical network features, thus reducing false positives. Finally, the fourth set of figures illustrates that for distant green fruits, the YOLOv8n model experiences missed detections, while the GreenFruitDetector, utilizing the MSOD architecture, offers superior performance in detecting distant, small-sized targets by better handling fine-grained information in the images.

## 4. Limitation and discussion

While the GreenFruitDetector model exhibits substantial performance gains in detecting green fruits within orchard environments, nevertheless, some challenges remain. The dataset currently lacks representations of nighttime scenes, adverse weather conditions, and other varied real-world scenarios. Additionally, the model is, at present, restricted to identifying only three specific types of green fruits, and its generalization to other fruit types or analogous characteristics remains to be validated. Thus, future research efforts should prioritize addressing these limitations.

The performance of the GreenFruitDetector object detection model has been evaluated and compared with other state-of-the-art models, with the results summarized in Table 10. It is not difficult to see that our model shows clear advantages in fruit detection within orchard environments compared to other algorithms.

**Table 10:** The performance comparison of existing model.

Model	Object	Average Precision (%)	Model Size(MB)	Inference(ms)
**GreenFruitDetector**	Korla fragrant pear	94.5	5.5	1.3
**YOLO-GEW [19]**	Yuluxiang pear	89.93	14.07	6.5
**YOLOMuskmelon [6]**	muskmelon	94.5	98.1	≈10.4
**LedNet [5]**	Apple	85.3	7.4	28

Additionally, the model is capable of detecting occluded objects and recognizing small targets. Combined with its lightweight design and high real-time performance, it holds potential for widespread applications in agricultural technology, such as pest detection and crop growth monitoring. Moreover, it is well-suited for applications in drone inspections and low-computation platforms.

## 5. Conclusions

This study investigates the detection of green fruits in orchard environments and proposes the GreenFruitDetector algorithm. The algorithm’s feature extraction capability is enhanced by introducing DCN. Furthermore, we design the MCAG-DC to replace the convolution in C2f and address the issues of leaf occlusion. Additionally, a Fusion-neck structure composed of SSFF, TFE, and MSOD modules is devised, offering better feature fusion capability and lightweight characteristics compared to baseline models, effectively enhancing detection capabilities for overlapping fruits and distant small targets. Finally, channel pruning is employed to compress the model, making it more lightweight while maintaining high accuracy.

The GreenFruitDetector model is compared with YOLOv3-Tiny, YOLOv5s, YOLOv7-tiny, the original yolov8 network and RT-DETR. Test results show that GreenFruitDetector outperforms these YOLO series models in terms of model size, parameter count, and detection accuracy. The model size is 3.0MB with 1.34M parameters. The accuracy of the enhanced model reached 94.5%, 84.4%, and 85.9% on the Korla Pear, Guava, and Green Apple datasets, respectively, representing improvements of 1.17%, 1.1%, and 1.77% over the baseline model.

In future work, we will introduce a more diverse dataset, including fruits of different varieties, maturity stages, and environmental conditions, enabling the model to adapt to a wider range of application scenarios. Finally, our targeted achievement is to deploy the model on embedded devices such as the Jetson Nano and integrate it with robotic arms to accomplish intelligent harvesting tasks.

## References

[1] Zhang Jun, Kang Ningbo, Qu Qianjin, Zhou Lianghuan, and Zhang Hongbo. ‘Automatic Fruit Picking Technology: A Comprehensive Review of Research Advances’. Artificial Intelligence Review 57, no. 3 (14 February 2024): 54. 10.1007/s10462-023-10674-2.

[2] Gao Guohua, Shuai Ciyin, Wang Shuangyou, and Ding Tao. ‘Using Improved YOLO V5s to Recognize Tomatoes in a Continuous Working Environment’. Signal, Image and Video Processing, 9 May 2024, 1–10. 10.1007/s11760-024-03010-w.

[3] Ukwuoma Chiagoziem C., Zhiguang Qin, Heyat Md Belal Bin, Ali Liaqat, Almaspoor Zahra, and Monday Happy N. ‘Recent Advancements in Fruit Detection and Classification Using Deep Learning Techniques’. Mathematical Problems in Engineering 2022 (31 January 2022): e9210947. 10.1155/2022/9210947.

[4] Koirala Anand, Walsh Kerry B., Wang Zhenglin, and Cheryl McCarthy. ‘Deep Learning–Method Overview and Review of Use for Fruit Detection and Yield Estimation’. Computers and Electronics in Agriculture 162 (1 July 2019): 219–34. 10.1016/j.compag.2019.04.017.

[5] Kang H., Chen C., 2020. Fast implementation of real-time fruit detection in apple orchards using deep learning. Computers and Electronics in Agriculture 168, 105108. 10.1016/j.compag.2019.105108

[6] Lawal O.M., 2021. YOLOMuskmelon: Quest for Fruit Detection Speed and Accuracy Using Deep Learning. IEEE Access 9, 15221–15227. 10.1109/ACCESS.2021.3053167

[7] Elmessery W.M., Maklakov D.V., El-Messery T.M., Baranenko D.A., Gutiérrez J., Shams M.Y., et al., 2024. Semantic segmentation of microbial alterations based on SegFormer. Front. Plant Sci. 15. doi: 10.3389/fpls.2024.1352935 38938642 PMC11208715

[8] Wan Shaohua, and Goudos Sotirios. ‘Faster R-CNN for Multi-Class Fruit Detection Using a Robotic Vision System’. Computer Networks 168 (26 February 2020): 107036. 10.1016/j.comnet.2019.107036.

[9] Pan Siyu, and Ahamed Tofael. ‘Pear Recognition in an Orchard from 3D Stereo Camera Datasets to Develop a Fruit Picking Mechanism Using Mask R-CNN’. Sensors 22, no. 11 (January 2022): 4187. 10.3390/s22114187. PMC9185418 35684807

[10] Liu Xiaoyang, Zhao Dean, Jia Weikuan, Ji Wei, Ruan Chengzhi, and Sun Yueping. ‘Cucumber Fruits Detection in Greenhouses Based on Instance Segmentation’. IEEE Access 7 (2019): 139635–42. 10.1109/ACCESS.2019.2942144.

[11] Liu Guoxu, Joseph Christian Nouaze Philippe Lyonel Touko Mbouembe, and Jae Ho Kim. ‘YOLO-Tomato: A Robust Algorithm for Tomato Detection Based on YOLOv3’. Sensors 20, no. 7 (January 2020): 2145. 10.3390/s20072145. PMC7180616 32290173

[12] Wang Wenhao, Shi Yun, Liu Wanfu, and Che Zijin. ‘An Unstructured Orchard Grape Detection Method Utilizing YOLOv5s’. Agriculture 14, no. 2 (February 2024): 262. 10.3390/agriculture14020262.

[13] Gu Bo, Wen Changji, Liu Xuanzhi, Hou Yingjian, Hu Yuanhui, and Su Hengqiang. ‘Improved YOLOv7-Tiny Complex Environment Citrus Detection Based on Lightweighting’. Agronomy 13, no. 11 (November 2023): 2667. 10.3390/agronomy13112667.

[14] Li Jianian, Liu Zhengquan, and Wang De. ‘A Lightweight Algorithm for Recognizing Pear Leaf Diseases in Natural Scenes Based on an Improved YOLOv5 Deep Learning Model’. Agriculture 14, no. 2 (February 2024): 273. 10.3390/agriculture14020273.

[15] Kang Rui, Huang Jiaxin, Zhou Xuehai, Ren Ni, and Sun Shangpeng. ‘Toward Real Scenery: A Lightweight Tomato Growth Inspection Algorithm for Leaf Disease Detection and Fruit Counting’. Plant Phenomics 6 (15 April 2024): 0174. doi: 10.34133/plantphenomics.0174 38629080 PMC11018486

[16] Sun Han, Wang Bingqing, and Xue Jinlin. ‘YOLO-P: An Efficient Method for Pear Fast Detection in Complex Orchard Picking Environment’. Frontiers in Plant Science 13 (4 January 2023). 10.3389/fpls.2022.1089454. PMC9846358 36684785

[17] Lyu Shilei, Li Ruiyao, Zhao Yawen, Li Zhen, Fan Renjie, and Liu Siying. ‘Green Citrus Detection and Counting in Orchards Based on YOLOv5-CS and AI Edge System’. Sensors 22, no. 2 (January 2022): 576. 10.3390/s22020576. 35062541 PMC8778674

[18] Nan Yulong, Zhang Huichun, Zeng Yong, Zheng Jiaqiang, and Ge Yufeng. ‘Faster and Accurate Green Pepper Detection Using NSGA-II-Based Pruned YOLOv5l in the Field Environment’. Computers and Electronics in Agriculture 205 (1 February 2023): 107563. 10.1016/j.compag.2022.107563.

[19] Ren R., Sun H., Zhang S., Wang N., Lu X., Jing J., et al., 2023. Intelligent Detection of Lightweight “Yuluxiang” Pear in Non-Structural Environment Based on YOLO-GEW. Agronomy 13, 2418. 10.3390/agronomy13092418

[20] Xu Zhi-Feng, Jia Rui-Sheng, Sun Hong-Mei, Liu Qing-Ming, and Cui Zhe. ‘Light-YOLOv3: Fast Method for Detecting Green Mangoes in Complex Scenes Using Picking Robots’. Applied Intelligence 50, no. 12 (1 December 2020): 4670–87. 10.1007/s10489-020-01818-w.

[21] Sun Meili, Zhao Ruina, Yin Xiang, Xu Liancheng, Ruan Chengzhi, and Jia Weikuan. ‘FBoT-Net: Focal Bottleneck Transformer Network for Small Green Apple Detection’. Computers and Electronics in Agriculture 205 (1 February 2023): 107609. 10.1016/j.compag.2022.107609.

[22] Zhu Xizhou, Hu Han, Lin Stephen, and Dai Jifeng. ‘Deformable ConvNets v2: More Deformable, Better Results’. arXiv, 28 November 2018. 10.48550/arXiv.1811.11168.

[23] Qi Yaolei, He Yuting, Qi Xiaoming, Zhang Yuan, and Yang Guanyu. ‘Dynamic Snake Convolution Based on Topological Geometric Constraints for Tubular Structure Segmentation’. arXiv, 18 August 2023. 10.48550/arXiv.2307.08388.

[24] Wang Lele, Zhao Yingjie, Liu Shengbo, Li Yuanhong, Chen Shengde, and Lan Yubin. ‘Precision Detection of Dense Plums in Orchards Using the Improved YOLOv4 Model’. Frontiers in Plant Science 13 (11 March 2022). 10.3389/fpls.2022.839269. PMC8963500 35360334

[25] Sun Lijuan, Hu Guangrui, Chen Chao, Cai Haoxuan, Li Chuanlin, Zhang Shixia, and Chen Jun. ‘Lightweight Apple Detection in Complex Orchards Using YOLOV5-PRE’. Horticulturae 8, no. 12 (December 2022): 1169. 10.3390/horticulturae8121169.

[26] Wang Dandan, and He Dongjian. ‘Channel Pruned YOLO V5s-Based Deep Learning Approach for Rapid and Accurate Apple Fruitlet Detection before Fruit Thinning’. Biosystems Engineering 210 (1 October 2021): 271–81. 10.1016/j.biosystemseng.2021.08.015.

[27] Shi Rui, Li Tianxing, and Yamaguchi Yasushi. ‘An Attribution-Based Pruning Method for Real-Time Mango Detection with YOLO Network’. Computers and Electronics in Agriculture 169 (1 February 2020): 105214. 10.1016/j.compag.2020.105214.

[28] Guava Dataset. 2024. Roboflow Universe. https://universe.roboflow.com/test-mm86f/guava-ycn8n (accessed on 2024-08-19).

[29] Häni N., Roy P., Isler V., 2020. MinneApple: A Benchmark Dataset for Apple Detection and Segmentation. IEEE Robotics and Automation Letters 5, 852–858. 10.1109/LRA.2020.2965061

[30] Liu Shu, Qi Lu, Qin Haifang, Shi Jianping, and Jia Jiaya. ‘Path Aggregation Network for Instance Segmentation’, n.d.

[31] Lin, Tsung-Yi, Piotr Dollar, Ross Girshick, Kaiming He, Bharath Hariharan, and Serge Belongie. ‘Feature Pyramid Networks for Object Detection’. In 2017 IEEE Conference on Computer Vision and Pattern Recognition (CVPR), 936–44. Honolulu, HI: IEEE, 2017. 10.1109/CVPR.2017.106.

[32] Li Xiang, Wang Wenhai, Wu Lijun, Chen Shuo, Hu Xiaolin, Li Jun, et al. ‘Generalized Focal Loss: Learning Qualified and Distributed Bounding Boxes for Dense Object Detection’. In Advances in Neural Information Processing Systems, 33:21002–12. Curran Associates, Inc., 2020. https://proceedings.neurips.cc/paper/2020/hash/f0bda020d2470f2e74990a07a607ebd9-Abstract.html.

[33] Kang Ming, Ting Chee-Ming, and Ting Fung Fung. ‘ASF-YOLO: A Novel YOLO Model with Attentional Scale Sequence Fusion for Cell Instance Segmentation’, n.d.

[34] Hou Qibin, Zhou Daquan, and Feng Jiashi. ‘Coordinate Attention for Efficient Mobile Network Design’. arXiv.org, 4 March 2021. https://arxiv.org/abs/2103.02907v1.

[35] Zhao Y., Lv W., Xu S., Wei J., Wang G., Dang Q., et al., 2024. DETRs Beat YOLOs on Real-time Object Detection.

